# Fibrosis Development in HOCl-Induced Systemic Sclerosis: A Multistage Process Hampered by Mesenchymal Stem Cells

**DOI:** 10.3389/fimmu.2018.02571

**Published:** 2018-11-05

**Authors:** Alexandre T. J. Maria, Karine Toupet, Marie Maumus, Pauline Rozier, Marie-Catherine Vozenin, Alain Le Quellec, Christian Jorgensen, Danièle Noël, Philippe Guilpain

**Affiliations:** ^1^IRMB, Montpellier University, INSERM, CHU Montpellier, Montpellier, France; ^2^Department of Internal Medicine–Multi-Organic Diseases, Saint-Eloi Hospital, Montpellier, France; ^3^Laboratory of Radiation Oncology Department, University Hospital of Lausanne (CHUV), Lausanne, Switzerland; ^4^Clinical Immunology and Osteoarticular Diseases Therapeutic Unit, Lapeyronie Hospital, Montpellier, France

**Keywords:** mesenchymal stem cells, systemic sclerosis, fibrosis, hypochlorite, oxidative stress, scleroderma, cell therapy, autoimmunity

## Abstract

**Objectives:** Skin fibrosis is the hallmark of systemic sclerosis (SSc) a rare intractable disease with unmet medical need. We previously reported the anti-fibrotic potential of mesenchymal stem cells (MSCs) in a murine model of SSc. This model, based on daily intra-dermal injections of hypochlorite (HOCl) during 6 weeks, is an inducible model of the disease. Herein, we aimed at characterizing the development of skin fibrosis in HOCl-induced SSc (HOCl-SSc), and evaluating the impact of MSC infusion during the fibrogenesis process.

**Methods:** After HOCl-SSc induction in BALB/c mice, clinical, histological and biological parameters were measured after 3 weeks (d21) and 6 weeks (d42) of HOCl challenge, and 3 weeks after HOCl discontinuation (d63). Treated-mice received infusions of 2.5 × 10^5^ MSCs 3 weeks before sacrifice (d0, d21, d42).

**Results:** HOCl injections induced a two-step process of fibrosis development: first, an ‘early inflammatory phase’, characterized at d21 by highly proliferative infiltrates of myofibroblasts, T-lymphocytes and macrophages. Second, a phase of ‘established matrix fibrosis’, characterized at d42 by less inflammation, but strong collagen deposition and followed by a third phase of ‘spontaneous tissue remodeling’ after HOCl discontinuation. This phase was characterized by partial fibrosis receding, due to enhanced MMP1/TIMP1 balance. MSC treatment reduced skin thickness in the three phases of fibrogenesis, exerting more specialized mechanisms: immunosuppression, abrogation of myofibroblast activation, or further enhancing tissue remodeling, depending on the injection time-point.

**Conclusion:** HOCl-SSc mimics three fibrotic phenotypes of scleroderma, all positively impacted by MSC therapy, demonstrating the great plasticity of MSC, a promising cure for SSc.

## Introduction

Skin fibrosis is the hallmark of systemic sclerosis (scleroderma, SSc), a rare intractable autoimmune disorder with high morbidity and mortality due to multi-organ involvement (1). Although precise etiology for SSc remains unknown, fibrosis development in the disease may originate from a complex interplay between environmental and intrinsic triggers–including oxidative stress- leading to tissue damage, immune response, endothelial cell and myofibroblast activation. The latter results in fibroblast proliferation and extracellular matrix (ECM) production (2–4). However, fibrogenesis is a dynamic process, with counteracting mechanisms involved to dampen immune activation or up-regulate tissue remodeling. So, different stages of tissue fibrosis may be observed, with variable participation of immune cell activation, angiogenesis or ECM synthesis by fibroblasts. Thus, targeting fibrosis in SSc may be challenged by the complexity of this multistage process, as well as the unpredictability of clinical evolution. Studies are still on going to help defining more precisely the clinical and biological status of SSc patients, hence refining therapy (5, 6).

Among new potential approaches for treating SSc, mesenchymal stem cells (MSCs)-based therapy stands as a promising lead (7). Indeed, these mesodermal multipotent progenitors can be easily isolated and expanded from virtually all tissues, and display both immunomodulatory and anti-fibrotic properties when infused (8). We recently demonstrated the feasibility and the therapeutic benefits of an approach based on MSCs in a preclinical murine model for SSc (9, 10). In this model, fibrosis is induced by repeated exposure to hypochlorite (HOCl), an oxidative agent administered by daily intra-dermal injections during 6 weeks. This model, mimicking early diffuse cutaneous SSc, is particularly useful to evaluate new therapeutic approaches. However, we still lack data on the development and the spontaneous evolution of fibrosis in HOCl-induced SSc (HOCl-SSc), as well as the impact of MSCs-based therapy along the process.

In this study, we first precisely characterized the fibrotic process induced by HOCl injections, as well as the evolution of fibrosis after discontinuation of HOCl challenge. Doing so, we further investigated the impact of MSC infusion, at each stage of the process, on skin fibrosis, inflammation and remodeling.

## Materials and methods

### Isolation and culture of mesenchymal stem cells

MSCs from BALB/c mice were isolated from bone marrow (BM). BM was flushed out from long bones and the cell suspension was plated in DMEM supplemented with 10% fetal bovine serum (FBS) (PAA Laboratories GmbH, Austria), 2 mM glutamine, 100 U/ml penicillin, 100 mg/ml streptomycin (Lonza, France). Cells were passaged till obtaining homogeneity for mesenchymal marker expression and lack of hematopoietic markers as analyzed by flow cytometry. They were used between passages 10 and 15.

### Flow cytometry analysis

Cells were harvested by treatment with 0.05% trypsin and 0.53 mM EDTA, and resuspended in PBS containing 0.1% BSA and 0.01% sodium azide. Cells were incubated on ice with conjugated antibodies against CD11b, CD44, CD45, CD73, and Sca1 (BD Pharmingen, France) or conjugated isotypic controls. Samples were analyzed on the FACS Canto II and analysis performed using the BD FACSdiva software (BD Pharmingen).

### Differentiation of MSCs

Differentiation of MSCs was induced as reported elsewhere (11). In brief, for adipogenesis, MSCs were plated at 10^4^ cells/cm^2^ in inductive medium and adipocytes characterized by presence of lipid droplets as visualized by Oil red O staining and expression of specific markers by RT-qPCR. Chondrogenic differentiation was induced by culture in micropellet and chondrogenesis was assessed by RT-qPCR. Osteogenesis was induced by culture at low density in osteogenic medium. Differentiation was assessed by RT-qPCR quantification of osteoblast markers and ECM mineralization visualized after staining with a 2% Alizarin Red S solution.

### T-cell proliferation assay

For T-cell proliferative experiments, 10^5^ splenocytes were stimulated with 1 μg/ml concanavalin A (conA; Sigma-Aldrich, France) in presence of different ratios of MSCs as already reported (11). After 3 days, cell proliferation was measured using the CellTiter-Glo™ luminescent cell viability assay (Promega, France).

### HOCl preparation

HOCl was generated extemporaneously by adding NaClO (9.6% as active chlorine) to KH_2_PO_4_ solution (100 mM, pH: 6.2), usually using a 1:100 ratio. The right amount of NaClO was adjusted to obtain the desired HOCl concentration, defined by the absorbance of the mixture at 292 nm (optical density between 0.7 and 0.9 read on a Nanodrop spectrophotometer, Thermoscientific). Stock solutions were stored at 4°C in the dark and NaClO was replaced every 3 weeks.

### Experimental design and animals

**Figure 1:**
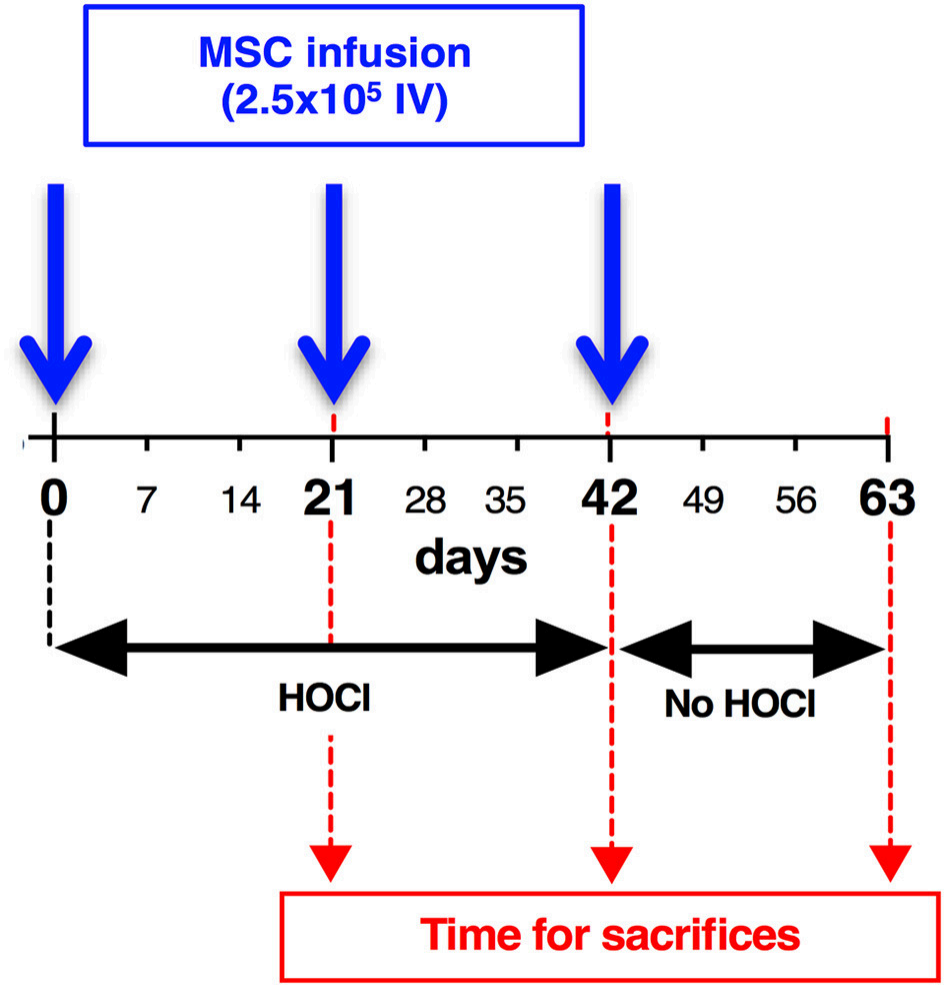
Development of skin fibrosis in HOCl-mice: experimental scheme. To induce systemic sclerosis, BALB/c mice underwent 6 weeks of daily HOCl intradermal injections, while control mice received PBS injections. Skin thickness was measured weekly during the experiment. After d42 (6 weeks), HOCl injections were abrogated, and groups of mice were kept in order to investigate skin thickness evolution till d63. Groups of mice were sacrificed at indicated time points (d21, d42, d63).

Six-week-old female BALB/c mice purchased from Janvier were housed and cared for according to the Laboratory Animal Care guidelines. Approval was obtained from the Regional Ethics Committee on Animal Experimentation before initiation of the study (approval APAFIS#5351-2016050919079187). All experiments were performed after final approval given by the French Ministry for Education, Higher Education and Research. Mice had their backs shaved the day before disease induction. Skin thickness was assessed with a caliper before disease induction and every week during the whole experiment by a blinded experimenter. As previously described, a total amount of 300 μl of freshly prepared HOCl was injected in two sites into the backs of the mice with a 29 G needle, 5 days a week for 6–9 weeks [d0 to d42 or day 63; (12)]. Control mice received PBS in the same conditions. MSCs-treated SSc-HOCl mice received an infusion of MSCs (2.5 × 10^5^ cells in 100 μl PBS), in the tail vein of the mice at indicated time points (d0, d21, d42). Groups of 7 to 10 mice were made for each condition (PBS-, HOCl- and MSCs-treated HOCl-mice). Three weeks after MSCs infusion, and after a 2-day recovery time without HOCl injections, animals were sacrificed, at indicated time points (d21, d42, or d63). Skin biopsies (6 mm punches) were taken on the backs of mice. Samples were stored at −80°C for RT-qPCR, ELISA and collagen content determination or fixed in 4% formaldehyde for histopathological analysis. Overall experimental scheme is shown in Figure 1, and representative pictures of mice during experimental procedure are shown in Supplementary Figure 1.

### Histopathology

Samples were embedded in paraffin and 5 μm thick sections were stained with Masson trichrome or immunostained with antibodies for α-sma (ab5694, Abcam, 1/100) TGFβ (ab66043, Abcam, 1 /100), Ki67 (SP6, VP-RM04, Vector laboratories, 1/200), CD3-epsilon (M-20, sc−1127, Santa Cruz Biotechnology, 1/250), F4/80 (MF4800, Invitrogen, 1/50), Pax5 (C-20, sc−1974, Santa Cruz Biotechnology, 1/250). Histological slides were scanned using Nanozoomer (Hamamatsu) or Pannoramic 250 Flash II (3DHistech) for immunofluorescence. Quantification of immunostaining was made using Definiens Tissue Studio software.

### RT-qPCR analysis

Samples (cells or skin biopsies) were crushed in RLT-buffer and total RNA was extracted using the RNeasy mini kit and Qiacube robotic workstation (Qiagen, France). 1 μg RNA was reverse transcribed using the Moloney Murine Leukemia Virus Reverse Transcriptase (M-MLV RT, Invitrogen, France). qPCR was performed on 20 ng cDNA using LightCycler 480 SYBRGreen I Master mix and real-time PCR instrument (Roche Applied Science, France). The following conditions were used: 95°C for 5 min; 40 cycles at 95°C for 15 s; 64°C for 10 s and 72°C for 20 s in a LightCycler 480 system (Roche diagnostics, France) and analyzed with the dedicated software. Primers were designed using the web-based applications Primer3 and BLAST (Table 1). Samples were normalized to mRNA expression of TATA binding protein *(Tbp)* gene for tissue samples or GAPDH for MSCs, and results were provided either as relative expression to the housekeeping gene using the formula 2^−Δ*Ct*^ or as fold change using the formula 2^−Δ*ΔCt*^.

**Table 1:** List of primers designed and used in RT-qPCR experiments.

**Gene abbreviation**	**Forward primer sequence (5^′^-3^′^)**	**Reverse primer sequence (5^′^-3^′^)**
*Ap*	GTGGTGGACGGTGAACGGGA	TCCACCGTGGGTCTCATGGC
*αSma*	AAGGCCAACCGGGAGAAAAT	AGCCAAGTCCAGACGCATGA
*Col1*	TGTTCAGCTTTGTGGACCTC	TCAAGCATACCTCGGGTTTC
*Col2B*	CTGGTGCTGCTGACGCT	GCCCTAATTTTCGGGCAT
*Col3*	CGGTGAACGGGGCGAAGCTGGTT	GACCCCTTTCTCCTGCGGCTCCT
*Col10*	TGCTGCCTCAAATACCCTTT	CAGGAATGCCTTGTTCTCCT
*Fabp4*	CGTAAATGGGGATTTGGTCA	TCGACTTTCCATCCCACTTC
*Gapdh*	GGTGCTGAGTATGTCGTGGA	GTGGTTCACACCCATCACAA
*Il1b*	TTTGACAGTGATGAGAATGACCTGTTC	TCATCAGGACAGCCCAGGTCAAAG
*Il6*	TGGGACTGATGCTGGTGACA	TTCCACGATTTCCCAGAGAACA
*Il10*	GGTTGCCAAGCCTTATCGGA	ACCTGCTCCACTGCCTTGCT
*Lpl*	TTTGGCTCCAGAGTTTGACC	GTCTTGCTGCTGTGGTTGAA
*Mmp1*	TTCAAAGGCAGCAAAGTATGGGCT	CCAGTCTCTTCTTCACAAACAGCAGCA
*Oc*	GCGCTCTGTCTCTCTGACCT	GCCGGAGTCTGTTCACTACC
*Pparγ*	AAGAGCTGACCCAATGGTTG	GGATCCGGCAGTTAAGATCA
*Runx2*	ACAGTCCCAACTTCCTGTGC	ACGGTAACCACAGTCCCATC
*Sox9*	AGGAAGCTGGCAGACCAGTA	CTCCTCCACGAAGGGTCTCT
*Tbp*	GGGAGAATCATGGACCAGAA	CCGTAAGGCATCATTGGACT
*Tgfβ1*	TGCGCTTGCAGAGATTAAAA	CTGCCGTACAACTCCAGTGA
*Timp1*	CTCCGCCCTTCGCATGGACATT	GGGGGCCATCATGGTATCTGCTCT
*Tnfα*	AGCCCACGTCGTAGCAAACCA	TGTCTTTGAGATCCATGCCGTTGGC

*Ap, alkaline phosphatase; aSma, alpha, smooth muscular actin; Col, collagen; Fabp, fatty acid binding protein; Gapdh, glyceraldehyde-3-phosphate deshydrogenase; Hmox, heme oxygenase; Il, interleukin; Lpl, lipoprotein lipase; Mmp, metalloprotease; Oc, osteocalcin; Ppar, peroxisome proliferator-activated receptor; Runx, Runt related transcription factor; Sox, SRY (sex determining region Y)-related HMG (high mobility group)-box gene; Tbp, TATA binding protein; Timp, tissue inhibitor of metalloproteinase; Tnf, tumor necrosis factor*.

### Collagen content in skin

Collagen content assay was based on the quantitative dye-binding Sircol method (Biocolor, Ireland). Skin biopsies were suspended in 2 ml of a 0.5 M acetic acid—pepsin (2.5 mg/ml) solution and dissociated using UltraTurrax (vWR, France). Collagen extraction was performed overnight at 4°C under stirring. Suspension was then centrifuged at 12,000 g for 10 min and 20 μl of each sample were added to 1 ml of Syrius red reagent. Tubes were rocked at room temperature for 30 min and centrifuged at 12,000 g for 10 min. The supernatants were discarded and tubes washed with 750 μl of ice-cold salt acid. After another 12,000 g centrifugation for 10 min, the collagen-dye pellets were suspended in 1 ml of 0.5 M NaOH. Optical Density (OD) was read at 555 nm on a microplate reader (Varioskan Flash, Thermo scientific) vs. a standard range of bovine collagen type I concentrations (supplied as a sterile solution in 0.5 M acetic acid). Results were expressed as collagen content in μg/mm^2^ of skin.

### Anti-topoisomerase 1 antibody elisa

Anti-topoisomerase 1 or anti-scl-70 antibodies were detected using scl-70 Ig ELISA kit (Abnova, Taiwan). In brief, 200 μl of 1:5 diluted sera were dispensed into scl-70 pre-coated wells and incubated at room temperature for 90 min. Goat total anti mouse IgG antibody, HRP conjugate (BD Biosciences, France), diluted 1:1,000 was then incubated for 60 min at room temperature. TMB substrate was incubated for 5 min and stopped with an equivalent amount of sulfuric acid. Absorbance was read at 450 nm under Varioskan Flash and results expressed as arbitrary unit (AU) for optical density (OD).

### Statistical analysis

All quantitative data were expressed as mean +/- SEM. Gaussian distribution of values was tested using the Shapiro-Wilk normality test. Data were then compared using Mann-Whitney's test for nonparametric values or Student's *t-*test for parametric values. When analysis included more than two groups, one-way ANOVA was used. All statistical analyses were performed using Prism 6 GraphPad software for Mac OS (California, United States). A *P* < 0.05 was considered significant.

## Results

### Induction of skin fibrosis under HOCl challenge is a two-step process with early proliferative and inflammatory stage and late constitution of ECM deposition

**Figure 2:**
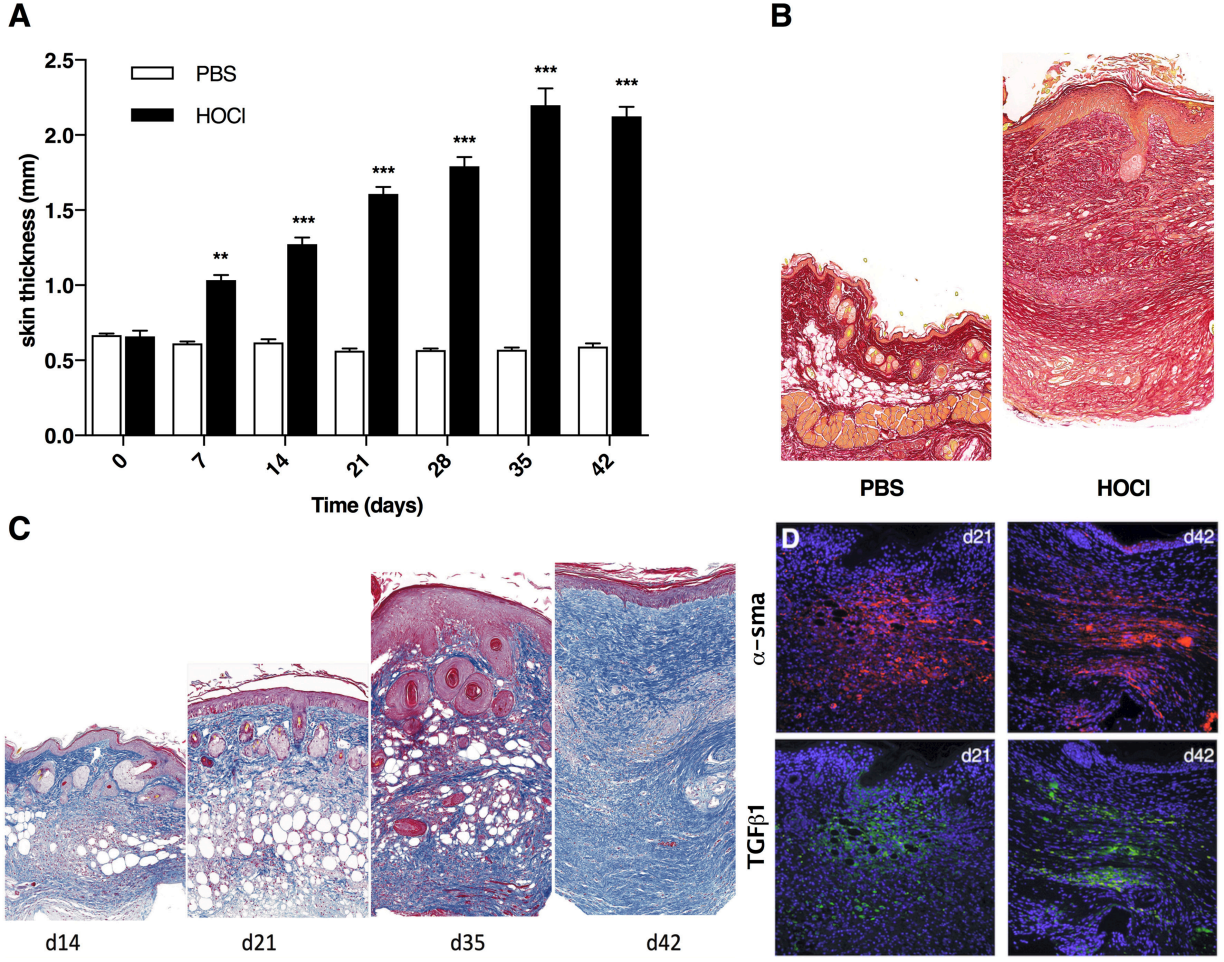
Development of skin fibrosis in HOCl-mice. **(A)** Skin thickness from PBS or HOCl-injected mice at different time-points during the induction of HOCl-SSc (d0 to d42) (*N* = 8 per group) **P < 0.001, ***P < 0.001. **(B)** Representative skin sections of PBS and HOCl mice at d42 (original magnification 10x; Sirius Red staining). **(C)** Representative skin sections at different time-points during the induction of HOCl-SSc (original magnification 10x; Masson Trichrome staining). **(D)** Immunostaining for αSma (in red) and Tgfβ1 (in green) in representative skin sections at d21 and d42.

As previously shown, the induction of skin fibrosis by daily HOCl injections was clinically characterized by progressive thickening of skin from d7 to d42 when compared with control PBS-mice (Figure 2A and Supplementary Figure 1). At d42, histology disclosed strong collagen deposition in skin from HOCl-mice compared with PBS mice, as shown by sirius red coloration (Figure 2B). Histological examination from sequential sacrifices revealed a discontinued process with two main stages. First, in the third week of experiment (d14–d21), we observed transparietal polymorphous cellular infiltrates; second, in the last week of experiment (d35–d42), these cellular infiltrates were gradually replaced by ECM deposition, resulting in disorganization of dermis and complete loss of hypodermic adipose tissue (Figure 2C). During the process, we noted a strong and steady expression of myofibroblastic markers α*Sma* and *Tgf*β*1* in dermis from SSc mice, with no obvious difference between d21 and d42 (Figure 2D).

**Figure 3:**
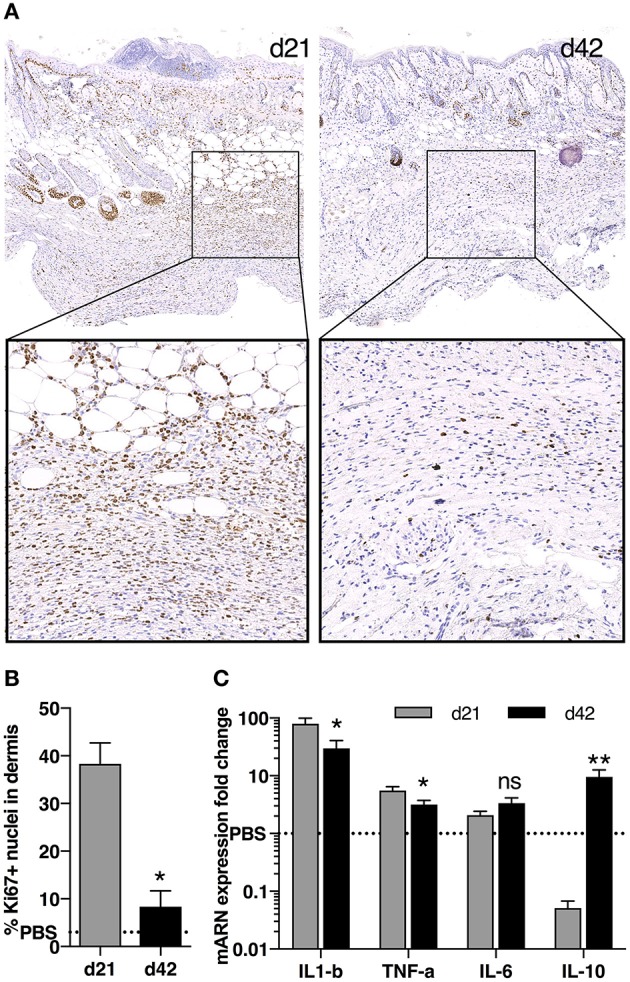
Cell proliferation and cytokine production in skin during fibrosis induction in HOCl-mice. **(A)** Immunostaining for nuclear Ki67 in representative skin sections at d21 and d42 (top panels: magnification 10x, bottom panels: top image box, 30x). **(B)** Percentage of Ki67^+^ proliferative cells in dermis as quantified on immunostaining in **A** using Definiens Tissue Studio software (*n* = 3 per group, *P < 0.05, **P < 0.01). **(C)** mRNA expression of *Il1b, Tnfa, Il6* and *Il10* in skin sections from HOCl-SSc mice at d21 and d42. Results are given as fold-change *vs*. control PBS-mice normalized at 1. *N* = 8 per group.

**Figure 4:**
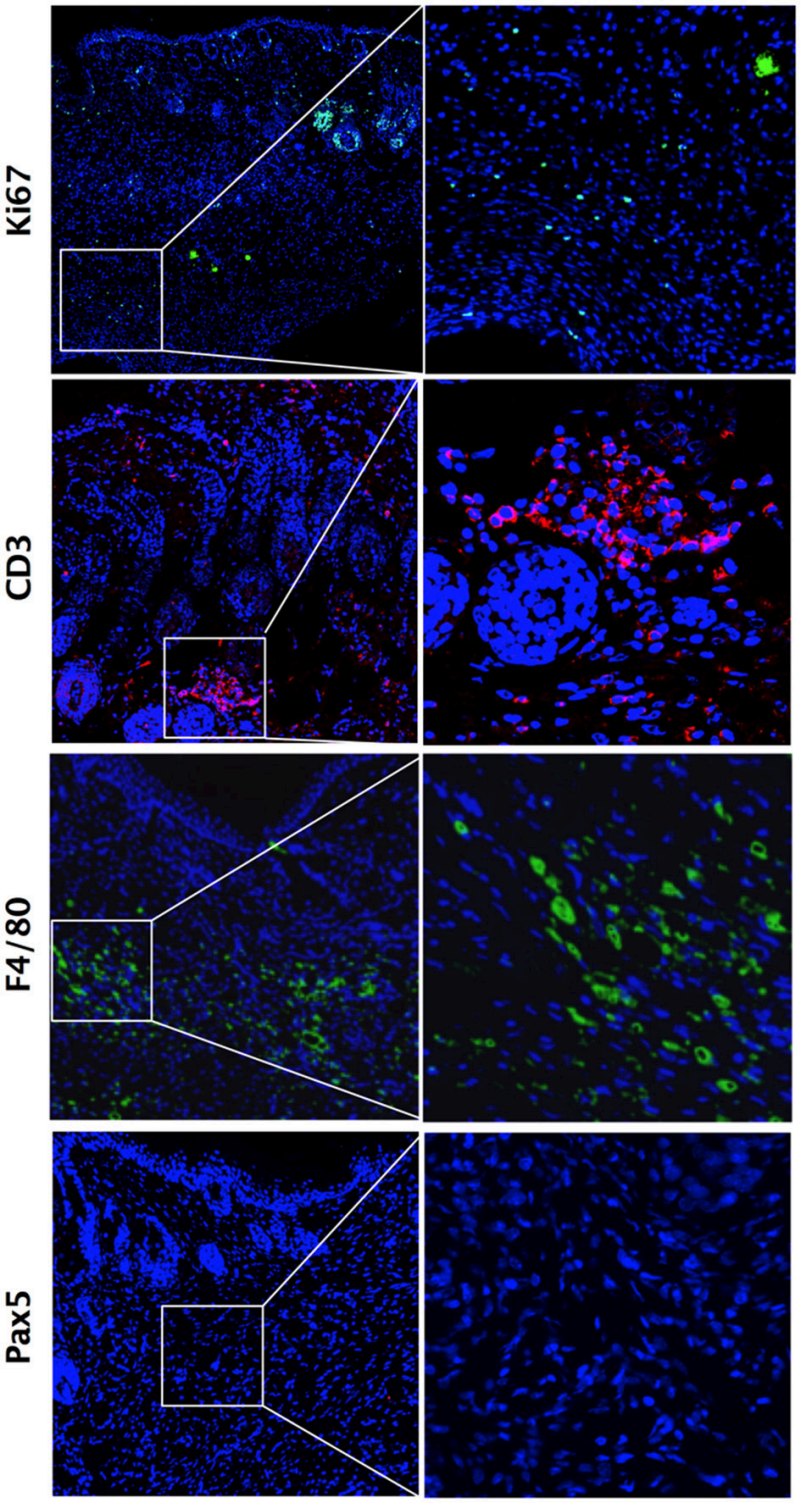
Inflammatory cell infiltrates within dermis during fibrosis induction in HOCl-mice. Immunostaining for Ki67^+^ proliferative cells, CD3^+^ T-lymphocytes, F4/80^+^ macrophages and Pax5^+^ B lymphocytes in representative skin sections at d21 from HOCl mice (original magnification 10x; enlargement 60x).

While comparing these two main time points (i.e., d21 and d42), we observed a higher number of proliferative cells in skin tissues at d21 compared with d42, as shown by Ki67 staining (Figures 3A,B). Looking at cytokine mRNA expression within the tissue, we noted a stronger expression of pro-inflammatory cytokines *Il1*β and *Tnf*α at d21 compared with d42, together with a lower expression of the anti-inflammatory cytokine *Il10* (Figure 3C). We therefore focused on d21, and further characterized these cellular infiltrates by immunostaining. We observed high number of CD3+ T-lymphocytes, of F4/80+ macrophages, but no Pax5+ B cells (Figure 4). Of note, we also noticed some cells co-expressing CD3 and Ki67 (data not shown).

Altogether, the analysis of skin fibrosis kinetics during the induction of HOCl-SSc revealed a two-step process, with an early cellular phase made of highly proliferative T-lymphocytes, macrophages and myofibroblasts, culminating at d21, and a late evolution toward paucicellular matricial fibrosis mainly characterized by ECM deposition at d42. Conversely, as previously reported, lung fibrosis development in this model is a continuous and progressive process, leading to tissue fibrosis at the end of the 6-wk experiment [(9) and Supplementary Figure 2). For these reasons and because skin fibrosis is the hallmark of SSc, we kept focused on skin fibrosis development in the present study.

### MSCs-based treatment efficiently reduces inflammation during the first stage of fibrosis constitution in HOCl-SSc

**Figure 5:**
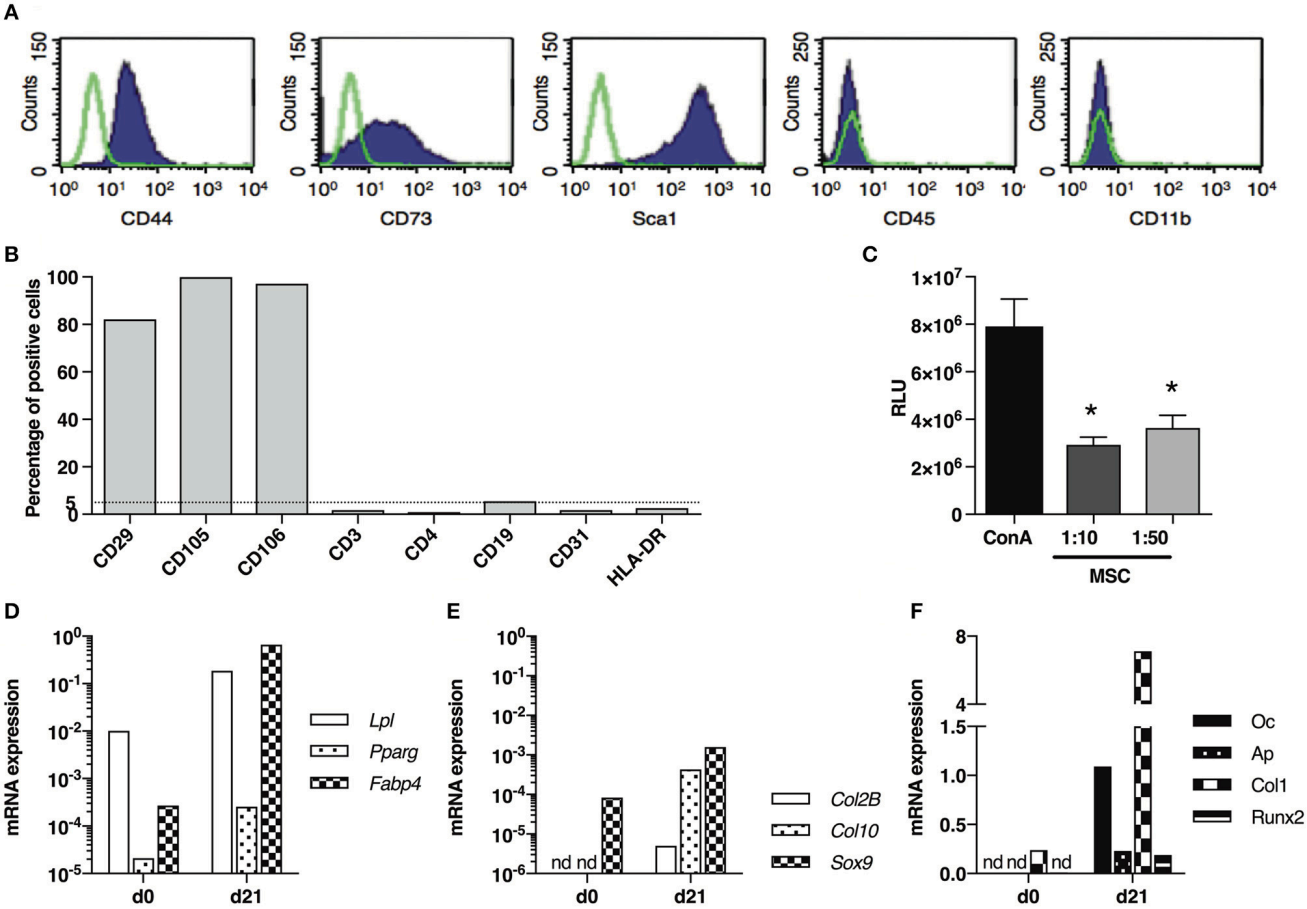
Phenotypical and functional characterization of BM-derived BALB/c MSCs. **(A)** Representative graph of flow-cytometry analysis of MSCs stained with antibodies against CD44, CD73, Sca1, CD45 and CD11b. **(B)** Percentage of MSCs stained with antibodies against CD29, CD105, CD106, CD3e, CD4, CD19, CD31 and HLA-DR in flow-cytometry analysis. **(C)** T cell proliferation assay expressed as relative luminescent units (RLU) for splenocytes stimulated with concanavalin A (ConA) and cultured in presence of MSCs (ratio 1:10 or 1:50; *n* = 3 per group) *P < 0.05. **(D)** mRNA expression of *Lpl, Pparg* and *Fabp4* in MSCs, normalized to *Gapdh* expression, before (d0) and after (d21) adipogenesis induction. **(E)** mRNA expression of *Col2B, Col10* and *Sox9* in MSCs, normalized to *Gapdh* expression, before (d0) and after (d21) chondrogenesis induction. **(F)** mRNA expression of *Oc, Ap, Col1* and *Runx2* in MSCs, normalized to *Gapdh* expression, before (d0) and after (d21) osteogenesis induction. Data are presented as average ± SEM. nd: not detected. *Ap*, alkaline phosphatase; *Col*, collagen; *Fabp*, fatty acid binding protein; *Lpl*, lipoprotein lipase; *Oc*, osteocalcin; *Ppar*, peroxisome proliferator-activated receptor; *Runx*, Runt related transcription factor*; Sox*, SRY (sex determining region Y)-related HMG (high mobility group)-box gene.

In a first experiment, we aimed at evaluating the anti-inflammatory impact of MSCs-based treatment administered during the first stage of fibrosis induction in the model (d0–d21). Therefore, we used MSCs isolated from BALB/c mice in a syngeneic approach. These cells were characterized by the expression of CD44, CD73, CD29, CD105, CD106, and stem cell antigen 1 (Sca1), and the absence of expression of the haematopoietic markers CD45, CD3, CD19, CD31, CD11b, and HLA-DR by cytometry analysis (Figures 5A,B). MSCs exerted immunosuppressive properties on the mitogen-induced proliferation of T-lymphocytes (Figure 5C) and were able to differentiate into adipocytes expressing lipoprotein lipase *(Lpl)*, peroxisome proliferator-activated receptor *(Ppar*γ*)*, fatty acid binding protein *(Fabp4)* (Figure 5D), chondrocytes expressing SRY (sex determining region Y)-related HMG (high mobility group)-box gene *(Sox9), Col2B, Col10* (Figure 5E) and osteoblasts expressing osteocalcin (*Oc)*, alkaline phosphatase *(Ap), Col1*, Runt related transcription factor (*Runx2;* Figure 5F).

**Figure 6:**
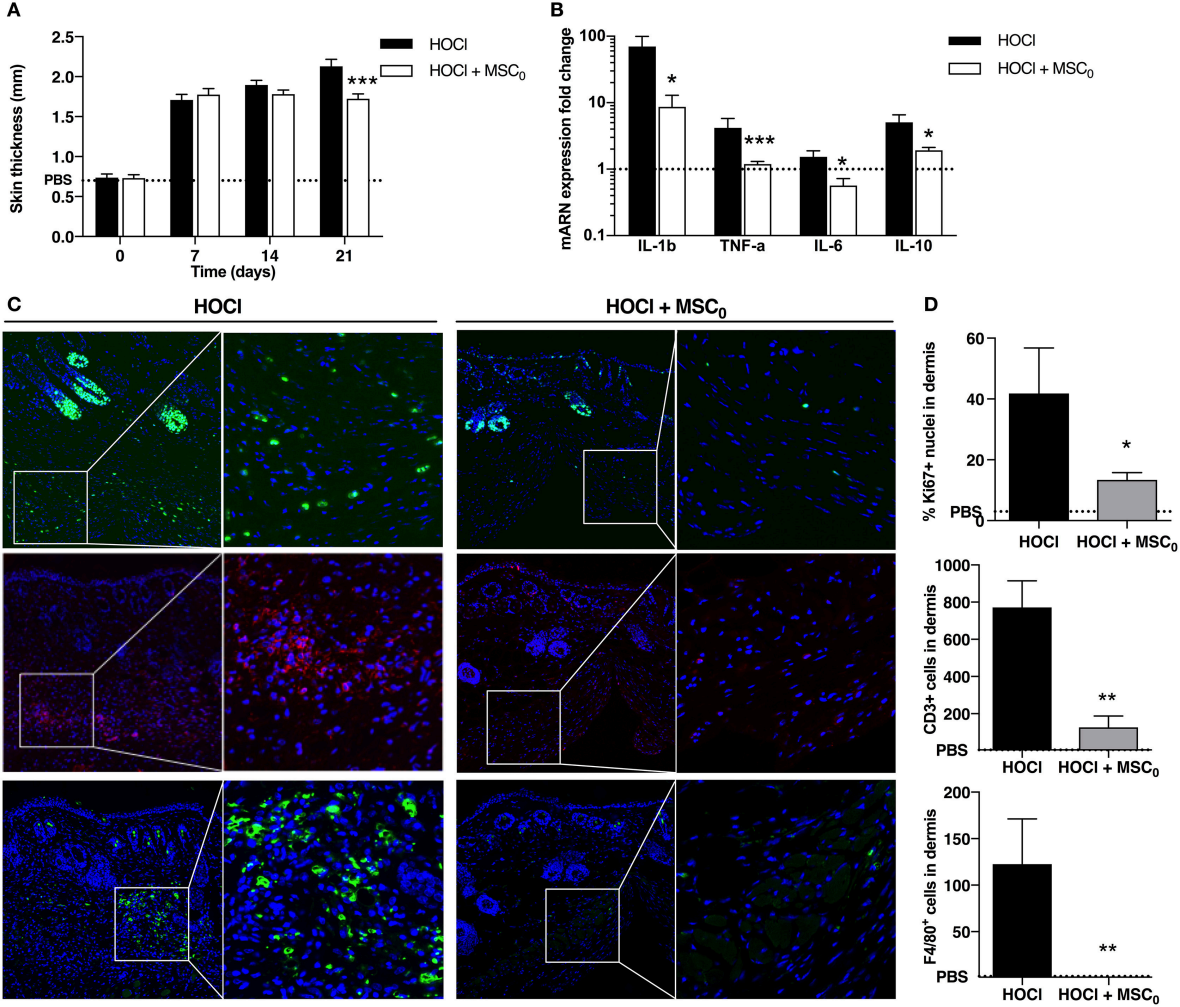
Anti-inflammatory effects of MSC treatment during fibrosis induction in SSc mice at day 21. **(A)** Skin thickness measured from d0 to d21 in control PBS mice, non-treated SSc mice and SSc mice treated with 2.5 × 10^5^ MSCs at d0 (MSC_0_). **(B)** mRNA expression of *IL1b, TNF-a, IL-6* and *IL-10* in skin at d21. Results are given as fold-change vs. control PBS-mice normalized to 1. **(C)** Nuclear immunostaining for proliferative cells using Ki67 (top panels, in green), CD3+ T-lymphocytes (middle panels, in red), and F4/80+ macrophages (bottom panels, in green), in representative skin sections at d21 from HOCl-SSc mice and MSCs-treated HOCl-SSc mice. Left: original magnification 10x; middle: part of left image 40x. **(D)** Quantification of immunostainings in **C** using Definiens Tissue Studio IF software (top: percentage of Ki67+ nuclei among all nuclei observed; middle and bottom: absolute number of CD3+ or F4/80+ cells; *n* = 4). *P < 0.05, **P < 0.01, ***P < 0.001, data are presented as mean ± SEM. *N* = 8 for HOCl-mice, *N* = 7 for MSCs-treated mice.

A unique dose of 2.5 × 10^5^ MSCs was administered intravenously before HOCl induction at d0, and mice were sacrificed 3 weeks later. A significant reduction of skin thickness was obtained at d21 in MSCs-treated SSc mice compared with untreated SSc mice (Figure 6A). Because inflammation was the highest at d21, we analyzed the effect of MSCs on inflammatory mediators in skin. MSCs treatment was associated with a significant decrease of *Il1*β*, Tnf*α*, Il6*, and *Il10* expression compared with untreated SSc mice (Figure 6B). Histological analysis revealed overall less cellular infiltrates in MSCs-treated mice, together with less Ki67 staining, strong decrease in CD3 staining and almost abolition of F4/80 staining (Figures 6C,D). MSC infusion also prevented specific anti-scl70 autoantibody production as detected in the serum (Supplementary Figure 3). On the whole, MSCs treatment during the first phase of HOCl-induced fibrosis prevented skin inflammation by down-regulating T-cell and macrophage immune response resulting in reduced cytokine and autoantibody production.

### MSCs treatment reduces myofibroblastic activation and collagen deposition during the second stage of fibrosis constitution in SSc mice

**Figure 7:**
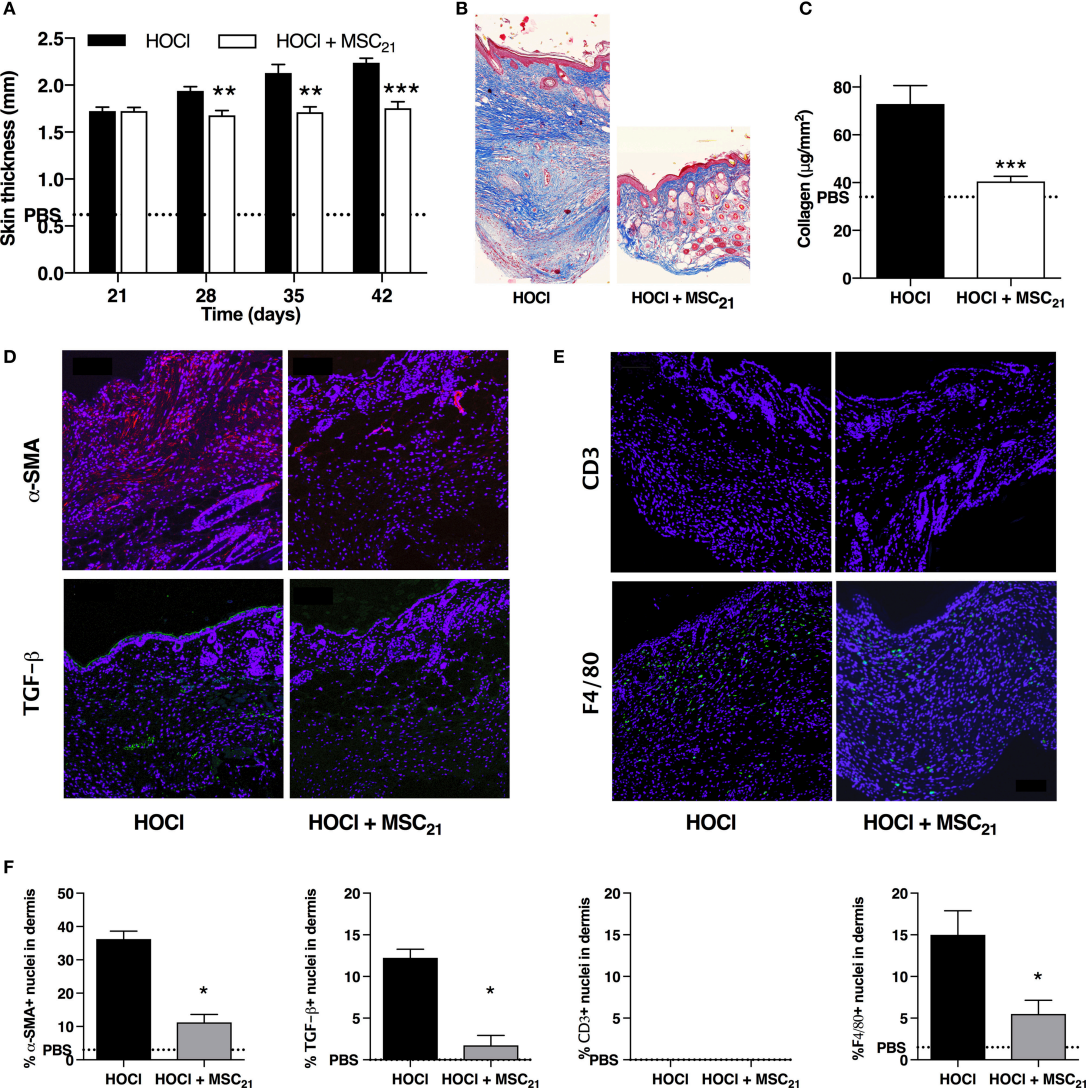
Anti-fibrotic and anti-inflammatory effects of MSCs treatment on skin fibrosis at day 42. **(A)** Skin thickness from d21 to d42 in HOCl-SSc mice, HOCl-SSc mice treated with 2.5 × 10^5^ MSCs at d21 (MSC_21_), or control PBS mice. *N* = 8 per group. **(B)** Representative skin sections at d42 from HOCl-SSc mice and MSCs-treated HOCl-SSc mice (original magnification 10x; Masson trichrome staining). **(C)** Collagen content in skin from HOCl-SSc mice and MSCs-treated HOCl-mice (MSC_21_). Mean level for control PBS mice are represented by a discontinued line. *N* = 8 per group. **(D)** Immunostaining with a-SMA (in red) and TGF-b1 (in green) in skin sections from HOCl-SSc mice (left) and MSCs-treated HOCl-SSc mice (MSC_21_, right) at d42. **(E)** Immunostaining with CD3 (in red) and F4/80 (in green) in skin sections from HOCl-mice (left) and MSC-treated HOCl-mice (MSC_21_, right) at d42. **(F)** Quantification of immunostainings in D and E (percentage of positive cells among all cells observed; *n* = 4 per group). *P < 0.05, **P < 0.01, ***P < 0.001 vs. HOCl-mice; data are presented as mean ± SEM.

We then investigated the effect of a single infusion of 2.5 × 10^5^ MSCs administered at d21 on the second phase of HOCl-SSc induction (d21–d42). MSCs treatment completely inhibited skin thickening during the whole period, resulting in significantly reduced skin thickness at d42 (Figure 7A and Supplementary Figure 1). At the end of the experiment, MSCs-treated mice exhibited reduced skin fibrosis characterized by less ECM deposition in Masson Trichrome staining (Figure 7B) and reduced total collagen content in skin (Figure 7C), almost reaching the level of control PBS mice. Immunostaining for α-Sma and Tgfβ confirmed less myofibroblastic activation in MSCs-treated mice compared with untreated mice (Figures 7D,F). Of note, fewer F4/80+ macrophages were also noted in skin from MSCs-treated mice compared with SSc mice, whereas no difference could be detected concerning CD3+ lymphocyte infiltrates, since there was almost none in SSc mice at d42 (Figures 7E,F). However, in this setting, we did not observe a reduction of anti-scl70 antibody production (Supplementary Figure 3).

### MSCs treatment after HOCl discontinuation activates tissue remodeling toward fibrosis clearance

**Figure 8:**
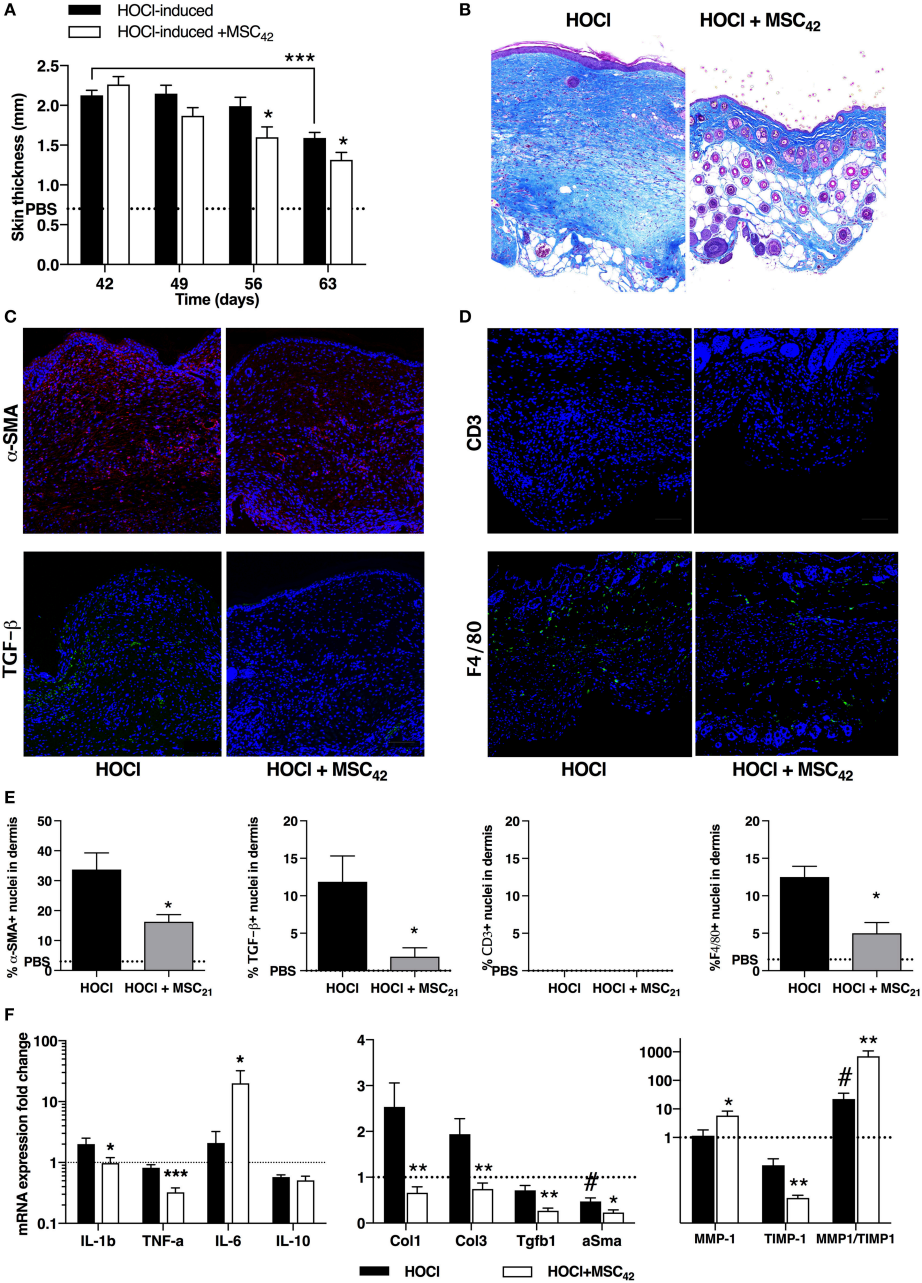
Tissue remodeling after fibrosis induction in HOCl-mice and effects of MSCs treatment at day 63. **(A)** Evolution of skin thickness after discontinuation of HOCl challenge in HOCl-induced mice, HOCl-SSc mice treated with 2.5 × 10^5^ at d42 (MSC_42_), or control PBS mice. **(B)** Representative skin sections from HOCl-SSc mice and MSCs-treated HOCl-mice (MSC_42_) (original magnification 10x, Masson trichrome staining). **(C)** Immunostaining with a-SMA (in red) and TGF-b1 (in green) in skin sections from HOCl-induced mice (left) and MSCs-treated HOCl-induced mice (right). **(D)** Immunostaining with CD3 (in red) and F4/80 (in green) in skin sections from HOCl-induced mice (left) and MSCs-treated HOCl-induced mice (right). **(E)** Quantification of immunostainings in C and D (percentage of positive cells among all cells observed; *n* = 4 per group). **(F)** mRNA expression of cytokines (*IL-1b, TNF-a, IL-6, IL-10*, left panel) fibrotic markers (*Col-1, Col-3, TGF-b1, a-SMA*, middle panel*)*, and remodeling parameters (*MMP-1* and *TIMP-1*, right panel*)* in skin sections from HOCl-induced mice compared with HOCl-mice treated with 2.5 × 10^5^ MSCs. Mean levels for control PBS-mice are represented by a discontinued line. *P < 0.05, **P < 0.01, ***P < 0.001 vs. HOCl-mice; #*P* < 0.05 vs. PBS-mice; data are presented as mean ± SEM. *N* = 8 per group.

In a last experiment, we aimed at investigating both the evolution of HOCl-SSc after HOCl discontinuation and the effect of MSCs treatment in this condition. Therefore, we administered a single infusion of 2.5 × 10^5^ MSCs at d42, stopped HOCl injections and sacrificed the mice 3 weeks later (d63). We first observed a spontaneous decrease in skin thickness in untreated HOCl-induced mice, significant in the last week of experiment (Figure 8A). MSCs-treated mice exhibited an earlier and stronger decrease in skin thickness compared with untreated HOCl-induced mice, significant in the last 2 weeks (Figure 8A). Histological analysis confirmed the strong anti-fibrotic effect of MSCs treatment, with an almost normal aspect of skin compared with untreated HOCl-induced mice (Figure 8B). Immunostaining for α-SMA and TGF-β corroborated these observations (Figures 8C,E), while mRNA expression for the main fibrotic markers confirmed a significantly reduced expression of *Col1, Col3, Tgf*β*1*, and α*-Sma* in MSCs-treated mice compared with untreated HOCl-induced mice (Figure 8F). Notably, levels of these four markers were found below the levels of control PBS-mice. Interestingly, untreated HOCl-induced mice also exhibited lower levels of α*-Sma* in comparison with control PBS mice. Concerning tissue inflammation, we noted residual F4/80 macrophage infiltrates in SSc mice, which was reduced in MSCs-treated mice (Figures 8D,E). In MSCs-treated mice we also observed a reduced expression of *Il1*β, and *Tnf*α, while *Il6* was increased and *Il10* remained unaffected (Figure 8F). Looking for changes in tissue remodeling, we noted a positive effect of MSCs treatment, associated with significant increased expression of metalloproteinase 1 (MMP1) and decrease of its main inhibitor tissue inhibitor of metalloproteinase 1 (TIMP1) vs. untreated HOCl-induced mice, resulting in a favorable MMP1/TIMP1 ratio in tissue (Figure 8F). Of note, untreated mice also exhibited a significantly higher ratio in comparison with PBS mice, or SSc mice sacrificed at d42 (data not shown). On the whole, HOCl discontinuation was responsible for tissue remodeling activation leading to progressive fibrosis clearance, a phenomenon significantly improved by MSCs therapy.

## Discussion

This study provides original data regarding skin fibrosis development in a preclinical inducible model for diffuse SSc. In this murine model, we demonstrated a multi-stage process leading to skin fibrosis under repeated exposure to HOCl.

First, during the first 3 weeks of the experiment, daily injections of HOCl were shown to trigger inflammation and led to transparietal cellular polymorphous infiltrates, with high proliferative rate, culminating at d21. These cellular infiltrates were mostly made of T-lymphocytes and macrophages, but also myofibroblasts. These observations are consistent with those made in SSc patients, notably during oedematous scleroderma, an inflammatory phenotype associated with early rapidly progressive diffuse SSc, where incisive treatments may be required (13, 14). In this condition, concordantly with what we observed, immune cells in skin tissue mainly include CD4+ T-lymphocytes and macrophages, with sparse B lymphocytes (15, 16). Macrophages are a well-known source of TGFβ secretion, this soluble mediator being the most critical trigger of fibroblast activation leading to ECM synthesis (3). Regarding cytokine expression, while IL1β and TNFα are associated with cell-mediated immunity, IL6 is also known to promote differentiation of fibroblasts into myofibroblasts (17) and to trigger collagen production (18). Moreover, at d21, we previously showed in this model upregulation of pro-inflammatory metalloproteases (MMP2 and MMP9, also known as gelatinases) and vascular endothelial growth factor [VEGF; (9)], which may activate angiogenesis and epithelial/endothelial-mesenchymal transition, contributing to myofibroblast activation and proliferation (19, 20). On the whole, the *primum movens* of fibrogenesis in HOCl-SSc is greatly related to inflammatory activation of skin tissue, leading to fibroblast proliferation, and preceding ECM synthesis.

In a second phase of HOCl-SSc induction, lasting from day 21 to day 42, we observed the progressive development of “matrix fibrosis,” characterized at d42 by paucicellular skin tissue, with panparietal ECM deposition made of disorganized collagen fibers. At the end of the 6-wk experiment, skin fibrosis in SSc mice was characterized by strong ECM deposition made of disorganized collagen fibers, along with strong thickening of epidermis and dermis, destructuring all skin layers. This state of “established fibrosis,” previously described in this model (9, 12) and responsible for skin thickening and stiffness, is close to what is usually observed in human SSc. Notably, as previously reported, tissue remodeling is low at d42, as indicated by decreased MMP1/TIMP1 ratio in tissue, contributing to impaired degradation of ECM components (9). Looking at the immune response, when comparing d42 to d21, we previously showed less inflammation and proliferation of cells, lower levels of inflammatory cytokines–except for IL-10 whose level increases during the 6-wk experiment. Of particular interest, dermal and hypodermal adipose tissue progressively disappeared during the whole process, as reported in the bleomycin model (21) and in human SSc (22), where it has been speculated that adipocyte attrition could even contribute to fibrogenesis through a putative adipocyte-to-fibroblast switch under PPAR-γ deregulation (23).

A spontaneous remission of SSc was observed once HOCl injections ceased, consistent with tissue remodeling activation and up-regulated MMP1/TIMP1 ratio. This third phase of fibrogenesis in HOCl-SSc had not been described before, and represents an original and interesting study model for SSc. As a matter of fact, clinical evolution in human scleroderma may be unpredictable, with patients first presenting with early and rapidly progressive diffuse SSc, but who may secondarily evolve with spontaneous improvement of skin fibrosis due to natural remodeling. Hence, this can somehow be disconcerting when considering therapeutic aspects, notably in the design of clinical trials (24, 25). Therefore, preclinical models reflecting disease heterogeneity and mimicking the different stages of fibrosis development are needed. In that perspective, HOCl-SSc, which reproduces three distinct phenotypes of scleroderma (i.e., early inflammatory phase, established matrix fibrosis, and spontaneous remodeling of tissue), seems particularly helpful to study new therapeutic approaches.

We previously reported the therapeutic benefits of a single IV infusion of 2.5 × 10^5^ MSCs, capable of preventing fibrosis development in a preventive approach, or hampering fibrosis extension in a curative approach. In the present study, considering the three steps of fibrosis development and receding in the model, we focused on the effects obtained 3 weeks after MSCs infusion (i.e., d21, d42, d63). We demonstrated beneficial effects of MSCs treatment for each therapeutic strategy, in terms of skin thickness and histological lesions improvement. However, benefits were associated with more specific mechanisms. In the first setting, when infused at d0, MSCs exhibited a pre-eminence of immunomodulatory properties and were able to efficiently prevent immune response, resulting in an extinction of cellular inflammatory infiltrates together with a strong decrease in cytokine production at d21. These immunomodulatory capacities of MSCs principally involve paracrine mechanisms with the secretion of various soluble factors (i.e., IDO, iNOS, PGE2) down-regulating the immune system (8). In the second setting, when infused at d21, MSCs treatment was associated with less myofibroblast activation, and less ECM deposition at d42. These effects were previously shown to depend primarily on TGFβ signaling abrogation by MSCs, resulting in lower expression of TGFβ, TGFβRII and phospho-SMAD in the tissue (9). In the last setting, when infused at d42, after discontinuation of HOCl injections, MSCs were shown to enhance tissue remodeling, leading to enhanced MMP1/TIMP1 ratio and a more rapid clearance of fibrotic lesions. Interestingly, lost adipose tissue seemed to be partially restored during the process, suggesting regenerative potential of MSCs. Of note, MSCs were also able to prevent autoantibody production when infused at d0, indicating a specific inhibition of B-cell activation toward plasma cells. However, we could not observe any effect when mice were treated at d21, maybe because of the *in vivo* half-life of IgG (about 3–4 weeks).

Altogether, we observed benefits of MSCs treatment whatever the setting of infusion, in three distinct conditions of fibrosis development, through immunosuppressive, trophic or regenerative properties. This indicated that not only do MSCs possess wide and pleiotropic capabilities, but they also show adaptability and versatility depending on the surrounding pathological environment. Actually, it has been shown that according to the signals in the vicinity (hypoxia, ischemia, cytokine secretion…), MSCs would be primed differently, and polarized into a specific phenotype [i.e., immunosuppressive, trophic…; (8, 26)]. This plasticity in response to specific injury makes MSCs-based therapy particularly interesting in treating SSc, considering the heterogeneity of the disease and the unpredictability of its evolution.

## Author contributions

AM participated in the design of the study, acquisition, analysis and interpretation of data, manuscript redaction and final approval. KT, MM, PR, and M-CV participated in acquisition and analysis of data, manuscript proofreading and final approval. AL and CJ participated in the design of the study, interpretation of data, manuscript preparation and final approval. DN and PG carried out the conception and design of the study, participated in analysis and interpretation of data, manuscript redaction and final approval.

### Conflict of interest statement

The authors declare that the research was conducted in the absence of any commercial or financial relationships that could be construed as a potential conflict of interest.

## Supplementary material

Supplementary Figure 1 **(A)** Skin thickness measurement during experiment (PBS mouse). **(B)** Skin at injection site on the mouse back during experiment (d34), in PBS injected-mice, HOCl-injected mice, HOCl-injected mice receiving MSC infusion at d21 (from left to right).

Supplementary Figure 2 **(A)** Representative lung sections at different time points during the induction of HOCl-SSc (Masson trichrome staining). **(B)** Lung sections from PBS mice and HOCl-SSc mice at d42 (red sirius staining).

Supplementary Figure 3 Levels of anti-scl70 antibodies in sera from PBS-healthy mice, HOCl-SSc mice and HOCl-SSc mice treated with MSC at d0 **(A)** or at d21 **(B)**. ^**^*P* < 0.01, ^***^*P* < 0.001; data are presented as mean ± SEM. *N* = 8 per group.
