# Supplementary figures and images for: Fibrosis Development in HOCl-Induced Systemic Sclerosis: A Multistage Process Hampered by Mesenchymal Stem Cells
Source: Front Immunol. 2018 Nov 5;9:2571. doi: 10.3389/fimmu.2018.02571 (PMC6230680; doi:10.3389/fimmu.2018.02571)

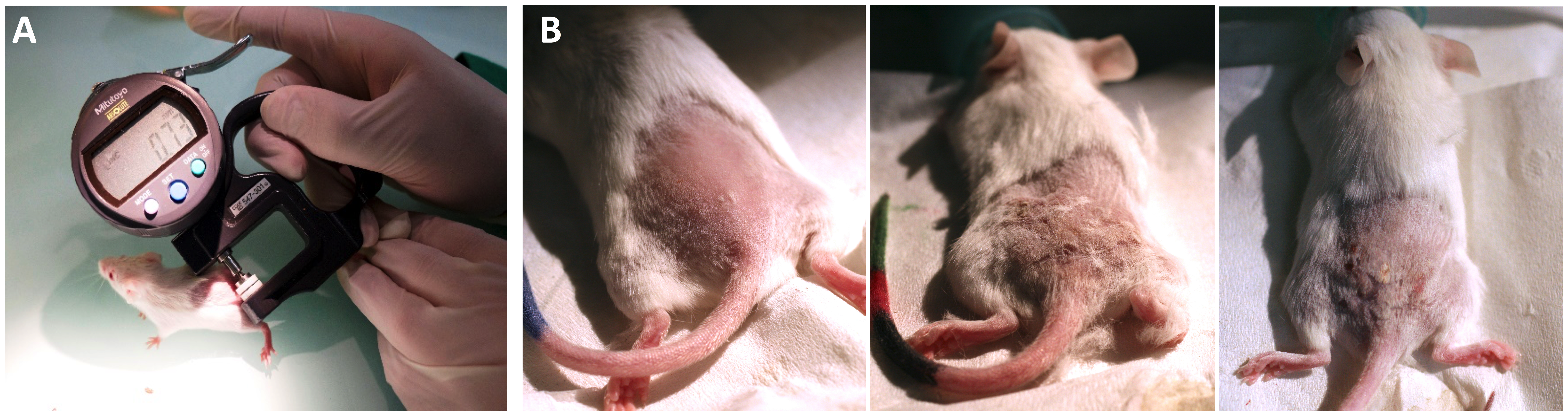

Supplement: Supplementary Figure 1 — (A) Skin thickness measurement during experiment (PBS mouse). (B) Skin at injection site on the mouse back during experiment (d34), in PBS injected-mice, HOCl-injected mice, HOCl-injected mice receiving MSC infusion at d21 (from left to right). [file Image_1.TIFF]

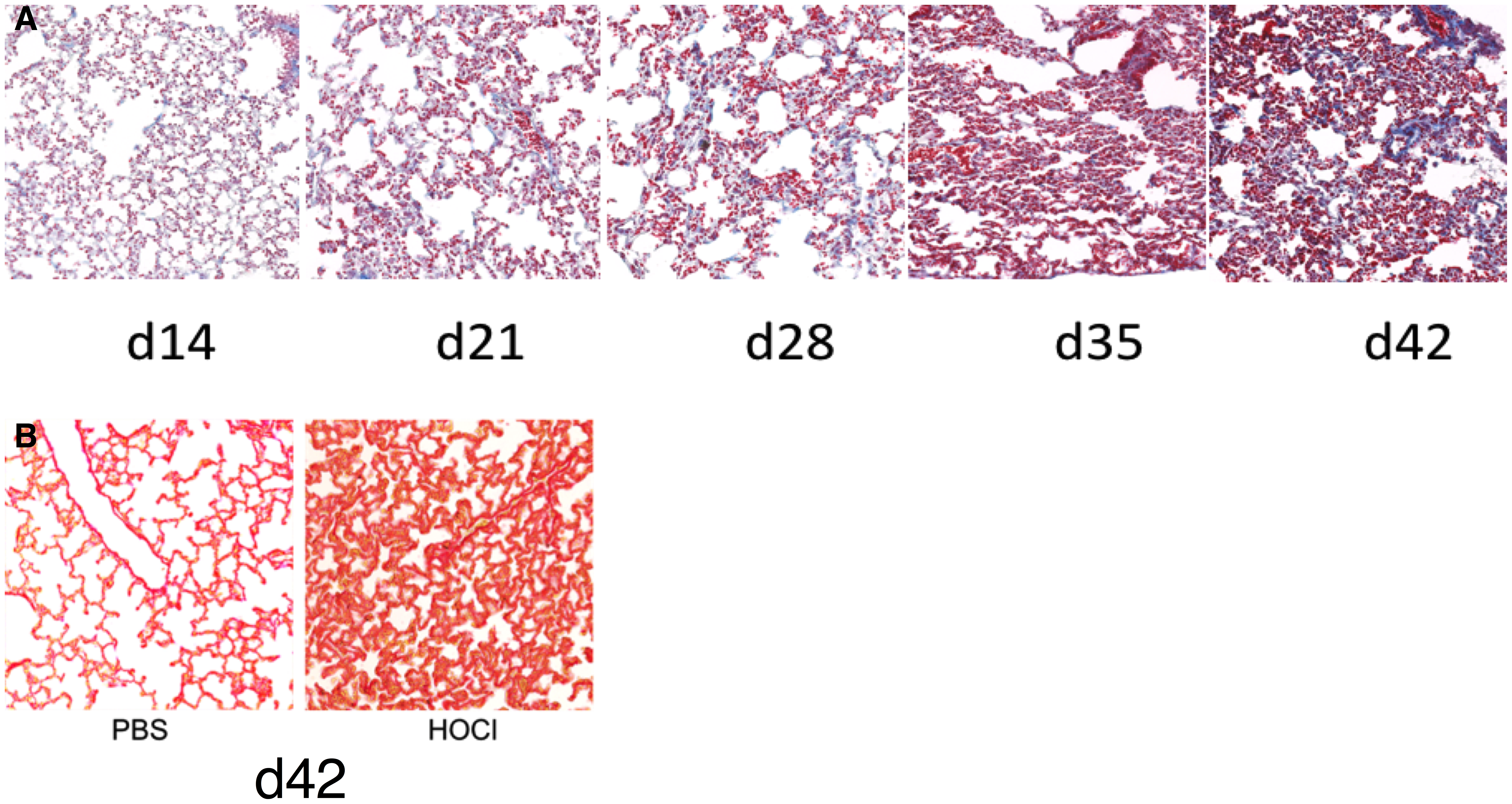

Supplement: Supplementary Figure 2 — (A) Representative lung sections at different time points during the induction of HOCl-SSc (Masson trichrome staining). (B) Lung sections from PBS mice and HOCl-SSc mice at d42 (red sirius staining). [file Image_2.TIFF]

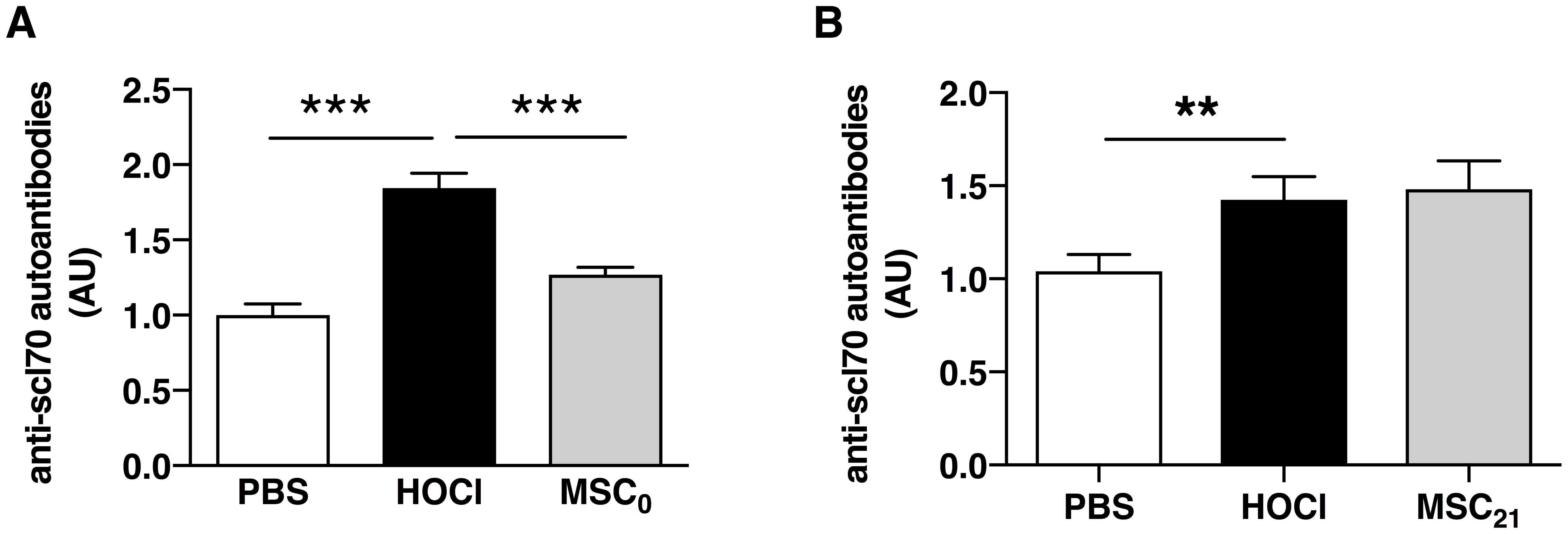

Supplement: Supplementary Figure 3 — Levels of anti-scl70 antibodies in sera from PBS-healthy mice, HOCl-SSc mice and HOCl-SSc mice treated with MSC at d0 (A) or at d21 (B). **P < 0.01, ***P < 0.001; data are presented as mean ± SEM. N = 8 per group. [file Image_3.TIFF]
